# Pipeline progress and portfolio management of the top 30 pharma companies over the past two decades

**DOI:** 10.1186/s40545-023-00612-6

**Published:** 2023-09-28

**Authors:** Melanie Büssgen, Marcel A. Büssgen

**Affiliations:** 1 Hamburg Center for Health Economics, University of Hamburg, 20354 Hamburg, Germany; 2 Leibniz University Hannover, Hannover, Germany

**Keywords:** Pharmaceutical industry, Strategy, Pipeline, Portfolio management, Clinical studies, Clinical trials, Pharma market

## Abstract

For pharmaceutical companies, it is essential to define their long-term corporate strategy. This especially involves the pipeline progress of pharmaceuticals and portfolio management. The objective of this study was to give a broad overview of study durations of the clinical trials from the top 30 pharmaceutical companies worldwide and to investigate what could possibly impact these study durations (e.g., indication areas, companies themselves, etc.) We worked with the clinicaltrials.gov database to examine the pipeline (phase 1–3) and portfolio (after regulatory approval) of the top 30 pharma companies worldwide over 20 years (from 2000–2020). We further calculated the study duration of each clinical study as the difference between the start date and end date. To analyze changes in our measure we estimated multiple linear regression to evaluate the impact of indication areas and companies on the study duration. Most of the clinical studies were conducted in the areas of ONCIM (*N* = 2720), and META (*N* = 1993). The indication with the highest study duration was ONCIM (on average 3.9 years per clinical study, SD: 0.8). Values for the study duration vary widely across companies. Mostly they range between 1 and 4 years (e.g., Merck Sharp & Dohme (MSD) on average 2.2 years per clinical study, SD: 1.0). Correlation analysis showed that study phases were positively correlated with the study duration (+ 0.36, *p* < 0.000), i.e., the higher the study phase, the higher the study duration. Furthermore, we found that indication areas influenced the study duration significantly (+ 0.17, *p* < 0.000). However, there were wide variations in effect sizes across indications. The results suggest that different indication areas influence the study duration to different extents. Pipeline progress and portfolio management differ widely between indications, companies and over the years. Research findings could help corporate strategy managers to make more informed decisions regarding their business development strategy.

**Supplementary Information:**

The online version contains supplementary material available at 10.1186/s40545-023-00612-6.

## Highlights

1. Pipeline and portfolio management differ widely between companies.
2. Most clinical studies were conducted in the area of oncology.
3. Study duration varies mostly between 1–4 years across companies.
4. Different indications influenced the study duration to different extents.

**Supplementary Information:**

The online version contains supplementary material available at 10.1186/s40545-023-00612-6.

## Background

Pharmaceutical companies are facing a range of challenges, including retaining their market share in highly fragmented markets with more and more players, taking a pioneering position in research to strengthen their reputation and launching pharmaceuticals to cover an urgent need [1–3]. To overcome these challenges and pave the way for a profitable future in times of economic recession, pharma companies need to re-evaluate their strategic portfolio management and constantly adapt it to current market trends as well as derive actions for accelerating their pipeline progress to be the first to bring the most promising drugs to market [4, 5]. With portfolio management centralizing all the information and processes necessary for identification, prioritization and managing of new pharmaceuticals, it acts as a central element for the success of a company [6]. In order to balance the trade-off between long study periods of clinical trials and steady cash flow at short intervals, it is essential that pharma companies develop and pursue a value-creating strategy that simultaneously gives them a competitive advantage [7].

Prior research has shown that the pipeline progress has substantially changed over the past decades. Only 12% of all pharmaceutical drugs are developed by the top 20 pharma companies which indicates that the market today is much more fragmented with more global players than before [8–10]. However, no statement can be made from this about the actual market shares. Further, it is widely known that portfolio management is the key to successful alignment of corporate strategy [11–13]. This means that for pharmaceutical companies, it is not only important to conduct clinical trials according to the highest scientific and ethical standards, but also to do so in a time-efficient manner in order to gain fast market access and thus a competitive advantage with the help of being the first mover. However, it has also been found that study durations increase despite industry efforts [14]. In addition, the average time that pharmaceutical companies want to keep their drugs on the market is increasing [15]. Innovation ambidexterity forces pharmaceutical companies to both continue to commercialize their existing products and to research and develop new products in order to be competitive in the long term and also generate short-term cash flow to pay for said research and development [16, 17].

The pipeline progress of new pharmaceuticals and the respective study durations of clinical trials have been subject of little research to date, especially investigating different areas of indication. We aim to fill this gap in the literature by analyzing the dynamics of the pipeline progress and the study durations of new pharmaceuticals across all 14 ATC indication areas for the top 30 pharmaceutical companies over the past two decades. Our findings can encourage discussions about a company’s portfolio management, identify optimization potentials in this regard and get pharma managers to rethink their company’s approach to pharmaceutical research and commercialization.

## Methods

### Data extraction

We extracted the information on clinical studies from clinicaltrials.gov for the following top 30 pharmaceutical companies (by revenue in 2020): AbbVie, Abbott, Allergan, Amgen, Astellas Pharma, AstraZeneca, Bausch & Lomb, Bayer, Biogen, Boehringer Ingelheim, Bristol-Myers Squibb, CSL Behring, Daiichi Sankyo, Eisai, Eli Lilly and Company, Gilead Sciences, GlaxoSmithKline, Johnson & Johnson, Merck Sharp & Dohme, Merck KGaA, Mylan, Novartis, Novo Nordisk, Otsuka, Pfizer, Roche, Sanofi, Takeda, Teva, and UCB. Clinicaltrials.gov is a web database that provides information about publicly and privately supported clinical studies (phase 1–4) on a wide range of diseases and conditions and is maintained by the National Library of Medicine (NLM) and National Institutes of Health (NIH) [18]. The data have been used for distantly related research before [19–21].

We examined the pipeline (phase 1–3) and the portfolio (after regulatory approval or in phase 4) of the top 30 pharma companies worldwide over 20 years. We restricted our sample to clinical studies that had their starting year between 2000 and 2020 to have a large, solid base for investigating the amount of clinical studies and study durations per indication and per pharma company.

### Statistical analysis

We operationalized pipeline progress and study duration in two ways: first, we calculated the amount of clinical studies conducted in each year in the past two decades (from 2000 to 2020) in each study phase (phase 1, phase 2, phase 3, and phase 4) and defined this as pipeline progress. We further differentiated this into all ATC level 1 code indication areas. Second, we calculated the length of clinical studies as the difference between the month of the staring year and the month of the end year and defined this as study duration. For our second outcome, we differentiated into all ATC level 1 code indication areas again, as well as by pharma companies. All 14 ATC level 1 code indication areas and their abbreviations can be found in Additional file 1.

Furthermore, we correlated the study duration with study phases. Because the investigated variables were not normally distributed (all *p*-values were highly significant using the Shapiro–Wilk test: study duration (*p* < 0.000), study phases (*p* < 0.000)), we employed correlation analysis according to Spearman.

### Regression model

To analyze changes in our measure, we estimated multiple linear regression to evaluate the impact of indication areas and company on the study duration.

$$\mathrm{Study} \, \mathrm{duration}= {\beta }_{0}+ {\beta }_{1}*\mathrm{indication} \, \mathrm{area}+ {\beta }_{2}* \mathrm{company}+ \varepsilon .$$

Study duration refers to our outcome of interest (dependent variable). Indication area (e.g., NEURO, URO, etc.) and company (e.g., AstraZeneca, Amgen, etc.) refer to our variables of interest (independent variables). $\varepsilon$ is an unobserved error term.

To avoid the eventuality of incorrect values being entered into the database, two researchers independently reviewed all records and sources several times, to verify the numbers given in the database.

All analyses were performed using STATA SE 16.

## Results

### Sample descriptives

Our final sample included 13,589 clinical studies of the top 30 pharma companies worldwide from 2000 to 2020. Differentiating the pipeline progress into each clinical study phase, we investigated 4998 phase 1 clinical studies, 2800 phase 2 clinical studies, 4219 phase 3 clinical studies, and 1572 phase 4 clinical studies. The amount of conducted clinical studies varied widely per indication area. While there were 2720 clinical studies in the ONCIM area and 1993 clinical studies in the META area, there were only 28 clinical studies in the HORM area. For the PARA area, we could not detect any clinical study from one of the top 30 pharma companies over the years. This was also checked and verified again with the red list (a German pharmaceutical register that lists ATC codes and manufacturers, among other things) [22]. Study durations varied widely across study phases and indication areas. While ONCIM clinical trials took on average 3.7 years, SENS clinical trials only took 1.0 year on average. Table 1 reports the sample descriptives.

**Table 1:** Sample descriptives

Variables	*N*	%	Study duration (in years)
Company	30		
Clinical trials	13,589	100	
Study phase			
Phase 1	4998	36.77	1.04
Phase 2	2800	20.60	2.32
Phase 3	4219	31.04	2.56
Phase 4	1572	11.56	1.87
Indications			
CARD	697	5.13	1.59
DERM	367	2.70	1.83
HAEM	264	1.94	2.13
HORM	28	0.21	1.84
INFEC	1364	10.04	1.88
META	1993	14.67	1.52
MUSCO	389	2.86	2.21
NEURO	1716	12.63	1.86
ONCIM	2720	20.02	3.72
PARA	0	0.00	
RESP	969	7.13	1.27
SENS	278	2.05	1.00
URO	359	2.64	1.82
VAR	225	1.66	1.32
Healthy volunteers (Phase 1)	2220	16.34	0.37

### Pipeline progress

Generally, we saw a dynamic towards an increase in the amount of clinical studies from 2005 to 2012. Especially the indication areas ONCIM, META, NEURO, and INFEC had a strong pipeline. However, we detected different peaks for each indication (e.g., ONCIM in year 2011 with 203 clinical studies, META in year 2010 with 164 clinical studies, NEURO in year 2007 with 161 clinical studies, and INFEC in year 2006 with 114 clinical studies).

On the other hand, the indication areas HORM, SENS, and HAEM had a rather weak pipeline. However, peaks were more or less constant for these indications and ranged around year 2008 (e.g., HORM in 2006, 2008, and 2010 with each 3 clinical studies, SENS in year 2008 with 32 clinical studies, and HAEM also in year 2008 with 33 clinical studies).

From year 2014 on, we saw a declining pipeline progress as the amount of conducted clinical studies decreased, even for research-strong indications, e.g., the amount of clinical studies decreased from 2014 to 2020 from 153 to 87 clinical studies in ONCIM, from 101 to 43 clinical studies in META, from 61 to 45 clinical studies in NEURO, and from 91 to 31 clinical studies in INFEC. We saw similar declines for indications with less clinical studies (e.g., SENS from 2014 to 2020 from 19 to 4 clinical studies).

Looking now at the conducted clinical studies per study phase, we saw that—here again—the amount of clinical studies increased from 2005 to 2012 and then decreased substantially across all study phases from 2012 to 2020, e.g., phase 1 from 336 to 204 clinical studies, phase 2 from 165 to 58 clinical studies, phase 3 from 249 to 112 clinical studies, and phase 4 from 68 to 24 clinical studies. Table 2 reports the descriptive statistics for the amount of clinical studies for the past two decades. For a differentiation of pipeline progress into each clinical study phase see Additional files 1, 2, 3.

**Table 2:** Clinical studies (*N*) per indication over the past two decades

Indications	2000	2001	2002	2003	2004	2005	2006	2007	2008	2009	2010	2011	2012	2013	2014	2015	2016	2017	2018	2019	2020
CARD	7	12	26	33	48	74	56	68	59	43	52	42	20	42	31	17	22	14	11	11	9
DERM	1	1	5	9	18	10	8	15	17	13	19	19	16	24	19	26	30	19	36	40	22
HAEM	1	3	11	4	16	16	19	21	33	25	15	10	11	10	15	8	15	9	11	7	4
HORM	1	2	0	1	2	1	3	1	3	1	3	0	2	2	2	1	0	1	0	0	2
INFEC	9	15	27	46	45	77	114	108	95	110	94	99	92	75	91	84	52	34	40	26	31
META	14	36	69	74	98	101	154	123	138	137	164	127	132	112	101	67	85	82	73	63	43
MUSCO	2	6	10	20	35	28	29	32	25	23	30	15	18	24	21	18	17	10	10	11	5
NEURO	15	25	52	88	136	110	154	161	150	132	128	106	78	62	61	52	36	34	54	37	45
ONCIM	20	25	56	84	96	147	192	196	200	207	193	203	159	150	153	115	129	102	112	94	87
PARA	0	0	0	0	0	0	0	0	0	0	0	0	0	0	0	0	0	0	0	0	0
RESP	5	7	31	26	38	54	52	76	69	65	83	74	85	65	62	49	39	45	17	17	10
SENS	3	3	10	13	17	21	17	19	32	17	29	16	15	14	19	10	7	3	4	5	4
URO	7	2	8	29	27	37	20	29	28	31	23	25	15	14	10	13	10	10	9	5	7
VAR	3	2	6	8	8	12	16	11	34	20	13	14	16	10	9	14	3	8	10	3	5

### Study duration

Generally, study durations varied widely across all indication areas. The indication with the highest study duration was ONCIM (on average 3.9 years per clinical study). Indications with the lowest study duration were SENS (on average 1.1 years per clinical study), RESP and VAR (both on average 1.5 years per clinical study). For all other indications, values varied between on average 1.6 years per clinical study (META) and on average 2.3 years per clinical study (HAEM and MUSCO). The average study duration in the ONCIM area is almost twice as long as that of URO clinical studies (on average 2.0 years per clinical URO study), and almost four times as long as that of SENS clinical studies (on average 1.1 years per clinical SENS study); see Table 3.

**Table 3:** Study duration (in years) per indication over the past two decades

Indications	2000	2001	2002	2003	2004	2005	2006	2007	2008	2009	2010	2011	2012	2013	2014	2015	2016	2017	2018	2019	2020	Mean (by indication)	SD (by indication)
CARD	3.7	2.5	1.9	2.4	1.4	1.3	1.4	1.3	1.3	2.0	1.6	1.5	1.3	1.6	1.9	1.0	1.8	1.9	2.2	1.6	1.5	1.8	0.6
DERM	0.6	2.8	2.0	2.0	1.6	1.2	1.6	1.6	0.9	1.0	2.8	1.4	2.6	1.2	1.7	1.2	1.5	2.3	2.4	2.4	2.1	1.8	0.6
HAEM	4.4	3.6	2.5	2.8	1.7	1.1	1.7	2.3	2.0	2.2	3.4	1.6	1.7	3.5	3.0	0.9	1.4	2.3	2.2	2.1	2.7	2.3	0.9
HORM	8.0	2.6		4.7	3.0	1.5	1.2	1.2	1.1	0.9	0.7		0.7	1.1	3.4	0.7		1.6			1.2	2.1	1.9
INFEC	3.5	3.1	2.8	3.0	2.5	2.1	1.7	1.8	1.8	1.7	1.6	1.9	1.6	1.8	1.5	1.9	2.0	2.0	1.5	2.0	2.1	2.1	0.5
META	2.4	2.1	1.7	1.4	1.7	1.6	1.5	1.2	1.3	1.3	1.3	1.3	1.5	1.2	1.8	1.9	1.5	1.8	2.1	2.1	1.9	1.6	0.3
MUSCO	2.1	4.1	3.1	1.2	1.8	2.0	1.6	1.8	2.9	1.9	2.4	1.6	2.4	3.0	2.6	2.2	2.2	2.7	1.8	3.7	2.0	2.3	0.7
NEURO	3.4	2.8	2.3	2.0	1.7	1.7	1.9	1.4	1.6	1.6	1.6	2.1	1.7	1.8	1.8	2.0	2.1	2.9	2.6	2.0	2.5	2.1	0.5
ONCIM	7.2	3.9	3.9	3.5	3.5	3.9	4.1	3.4	3.7	3.6	3.3	3.2	3.7	3.5	3.7	4.1	3.6	3.9	3.8	4.3	4.3	3.9	0.8
PARA																							
RESP	3.4	2.2	1.4	1.7	1.6	1.5	1.6	0.9	0.7	1.2	0.9	1.1	1.4	1.3	1.4	1.3	1.3	1.4	1.3	2.0	1.5	1.5	0.5
SENS	0.3	0.8	0.7	1.0	1.0	0.7	0.7	0.7	0.7	1.1	0.8	1.4	0.8	1.3	1.2	2.5	0.9	0.6	1.1	2.7	1.6	1.1	0.6
URO	2.8	4.5	2.9	1.7	1.9	2.4	1.8	1.6	1.7	2.0	1.2	1.7	1.5	1.9	2.2	1.2	1.8	2.1	0.8	1.4	2.4	2.0	0.7
VAR	2.3	0.9	1.9	1.6	2.6	1.7	1.8	0.7	0.8	0.4	2.0	2.7	0.9	0.7	0.8	1.4	2.0	1.8	1.2	1.2	1.3	1.5	0.6
Mean (by year)	3.4	2.8	2.3	2.2	2.0	1.7	1.7	1.5	1.6	1.6	1.8	1.8	1.7	1.8	2.1	1.7	1.8	2.1	1.9	2.3	2.1		
SD (by year)	2.1	1.1	0.8	1.0	0.7	0.7	0.7	0.7	0.9	0.8	0.9	0.6	0.8	0.9	0.8	0.9	0.6	0.8	0.8	0.9	0.8		

Values for the study duration also vary across companies. Mostly they range between 1 and 4 years (e.g., GlaxoSmithKline on average 1.9 years per clinical study, Merck Sharp & Dohme (MSD) on average 2.2 years per clinical study, Novartis on average 2.9 years per clinical study). Companies with the lowest mean study durations were Mylan (on average 0.9 years per clinical study) and Novo Nordisk (on average 1.1 years per clinical study), and companies with the highest mean study duration were Gilead Sciences and Roche (both on average 3.3 years per clinical study). The fastest clinical study started in year 2013 with a study duration of 0.1 years (CSL Behring) and the longest in year 2000 with a study duration of 13.3 years (Gilead Sciences). When comparing the average study duration of 2000 vs. the average study duration of 2020, we see that the study duration has decreased substantially over time (2000: on average 4.3 years vs. 2020: on average 2.0 years). However, there even was a larger decrease around 2013 (on average: 1.8 years), but from then on, a slight increase in study duration occurred again. Nevertheless, the study duration has decreased by 100% from 2000 to today; see Table 4.

**Table 4:** Study duration (in years) per company over the past two decades

Company	2000	2001	2002	2003	2004	2005	2006	2007	2008	2009	2010	2011	2012	2013	2014	2015	2016	2017	2018	2019	2020	Mean (by company)	SD (by company)
AbbVie							2.0	3.6	2.7	5.1	0.6	0.6	6.0	2.5	3.7	2.4	4.1	3.0	3.6	3.2	2.2	3.0	1.4
Abbott	6.0		4.3	3.3	4.4	2.1	1.8	2.6	1.9	1.6	1.9	2.0	1.9	3.0	3.5		2.5					2.9	1.2
Allergan			1.2	1.0	4.0	2.9	2.0	1.9	1.1	1.6	1.2	1.9	0.8	1.6	1.8	2.7	0.8	2.6	2.1	2.6	1.4	1.8	0.8
Amgen			2.4	1.7	3.3	3.1	3.0	3.4	3.3	2.8	2.9	1.7	2.2	2.4	1.9	2.7	1.9	3.9	3.2	3.1	2.9	2.7	0.6
Astellas Pharma	0.6		2.3	3.2	1.9	2.8	1.9	1.7	1.3	1.5	1.1	2.0	2.0	3.0	2.4	0.3	1.0	0.6	0.2	0.9	0.7	1.6	0.9
AstraZeneca	5.5	3.0	1.5	2.8	2.5	2.2	2.5	1.8	1.2	0.8	1.1	0.9	1.2	2.1	2.2	2.6	1.4	2.5	2.7	2.8	3.0	2.2	1.0
Bausch & Lomb	1.5		0.8	2.7	1.8	1.8	0.7	0.9	0.9	0.9	1.5	2.5	2.2	1.4	1.0	1.3	0.9	0.6	1.2	1.7	1.5	1.4	0.6
Bayer	6.8	2.7	2.7	1.5	1.3	2.1	1.7	2.6	3.1	2.9	1.6	2.2	3.4	2.6	2.1	2.1	3.6	1.9	1.5	2.1	2.2	2.5	1.1
Biogen			5.0	3.5	1.7	1.4	5.4	1.7	2.0	2.0	0.9	1.2	1.5	1.7	2.1	1.9	1.1	2.1	3.1	1.8	1.6	2.2	1.2
Boehringer Ingelheim	2.6	2.2	1.5	1.3	1.0	3.3	1.0	4.0	6.1	4.4	3.5	2.9	1.1	1.6	1.0	1.0	1.5	1.0	1.8	1.1	1.4	2.2	1.4
Bristol-Myers Squibb	6.9	3.0	3.1	2.5	1.5	3.3	2.1	3.4	2.2	2.5	1.7	2.9	1.8	2.6	1.3	3.0	2.5	2.3	1.0	2.6	0.9	2.5	1.2
CSL Behring						2.9	0.6	0.3	1.4	0.8	1.7	2.1	1.2	0.1	3.8				3.7	2.8	0.6	1.7	1.2
Daiichi Sankyo	4.5	3.8	2.5	2.6	1.8	1.6	1.4	1.0	1.6	2.8	1.7	3.5	1.7	1.5	0.6	0.8	1.6	1.7	3.4	0.7	2.4	2.1	1.0
Eisai			6.9	3.5	2.9	3.1	3.4	2.1	1.9	2.4	1.4	3.0	2.6	1.4	1.0	1.3	1.7	2.2	1.4	3.8	2.2	2.5	1.3
Eli Lilly and Company	8.1	2.5	2.8	2.2	2.2	3.4	2.7	2.5	3.0	2.4	2.1	1.6	1.7	1.1	2.1	1.6	1.3	1.0	1.4	1.4	1.3	2.3	1.4
Gilead Sciences	13.3		1.9	3.3	2.8	5.7	4.6	4.9	2.7	3.3	2.4	2.2	2.4	1.8	1.9	2.3	3.1	2.1	1.8	1.8	1.5	3.3	2.5
GlaxoSmithKline	5.2	3.1	2.1	2.3	2.4	1.4	1.5	1.2	1.3	1.4	1.2	1.5	1.2	1.1	1.5	1.1	1.5	1.9	1.7	2.8	3.1	1.9	1.0
Johnson & Johnson	1.7	2.8	1.6	0.8	1.6	1.3	2.7	1.7	1.5	1.7	1.2	0.9	1.2	1.1	1.6	1.8	1.3	1.8	1.6	2.1	1.9	1.6	0.5
Merck Sharp & Dohme	2.2	2.4	1.1	1.2	1.3	1.7	1.6	1.5	1.1	1.6	1.5	1.6	1.8	1.3	2.4	1.8	3.6	3.8	4.0	3.7	4.2	2.2	1.0
Merck KGaA				0.8	2.7		3.2	3.2	2.3	3.8	4.3	2.5	3.8	3.6	1.3	3.3	2.4	2.1	0.9	1.6	1.3	2.5	1.1
Mylan		2.6	0.7	0.6	0.5	0.6	1.3	0.8	1.1	0.9	1.0	0.6	1.2	0.8	0.7	0.9	0.5	0.8	0.8	0.8		0.9	0.5
Novartis	6.9	3.3	3.0	3.3	1.7	2.2	2.5	2.1	2.5	2.6	2.8	2.9	2.6	3.4	2.5	2.8	2.5	2.2	3.5	2.9	3.3	2.9	1.0
Novo Nordisk	0.8	1.3	0.9	0.8	1.4	0.8	0.8	1.0	0.9	0.9	0.8	0.7	1.4	1.1	1.4	1.4	1.3	1.5	1.4	2.0	1.3	1.1	0.3
Otsuka	3.3		2.0	3.9	2.3	2.2	2.4	1.7	2.7	2.3	2.7	2.0	1.5	1.7	1.8	2.7	0.7	2.9	2.3	1.0	1.2	2.2	0.8
Pfizer	3.0	3.7	3.5	2.2	1.9	2.2	1.6	1.4	1.5	1.2	1.0	1.1	1.6	1.3	1.3	1.7	1.5	1.6	1.3	1.0	1.1	1.7	0.8
Roche	4.8	4.8	5.3	4.4	3.0	3.6	3.6	2.6	2.7	2.6	2.6	2.4	2.2	2.4	3.1	3.3	2.6	3.3	2.7	3.1	3.5	3.3	0.9
Sanofi	3.5	3.1	2.4	2.0	2.2	2.0	2.8	1.8	1.7	1.5	1.9	2.1	1.7	1.6	1.5	1.6	1.6	1.7	1.4	1.7	3.5	2.1	0.6
Takeda	3.3	5.8	2.6	2.0	1.0	1.1	1.5	1.3	1.5	1.6	2.1	1.5	1.7	1.2	1.4	1.7	1.6	1.4	0.7	1.0	1.5	1.8	1.0
Teva	1.0	0.3	0.3	0.1	0.4	1.0	0.2	0.6	1.9	2.4	3.7		1.5	1.1	0.8	0.8	1.4	0.5	2.6	2.8	2.1	1.3	1.0
UCB	3.5	5.4	1.8	2.4	2.4	2.3	1.9	2.0	2.3	3.3	2.8	2.9	1.5	1.4	1.9	8.5	1.2	2.9	1.8	2.6	3.0	2.8	1.6
Mean (by year)	4.3	3.1	2.5	2.2	2.1	2.3	2.1	2.0	2.0	2.2	1.9	1.9	2.0	1.8	1.9	2.1	1.8	2.0	2.0	2.1	2.0		
SD (by year)	2.88	1.28	1.49	1.09	0.93	1.05	1.13	1.06	1.02	1.05	0.92	0.77	0.98	0.80	0.83	1.46	0.89	0.90	1.00	0.89	0.94		

Correlation analysis using Spearman’s rank correlation coefficient showed that study phases were positively correlated with the study duration (0.36, *p* < 0.000), i.e., the higher the study phase, the higher the study duration. However, it should be noted that pharma companies which conduct many phase 3 studies have higher mean study duration values than companies that primarily conduct only phase 1 studies and, for example, do not send their drugs into the following study phases because the expected study outcome does not promise success.

### Regression results

Our descriptive results were confirmed by our regression results. In general, we saw that indication areas influenced the study duration significantly (+ 0.169, *p* < 0.000); see Table 5. However, there were wide variations in effect sizes. Coefficients mostly ranged around 1.5, e.g., DERM: 1.37, *p* < 0.000, META: 1.32, *p* < 0.000, and HORM: 1.57, *p* < 0.000 (see Additional files 1, 2, 3). The results suggest that different indication areas influence the study duration to different extents. While ONCIM clinical studies took 3.27 years longer (*p* < 0.000) compared to healthy individuals/phase 1 clinical studies, SENS clinical studies only took 0.56 years longer than our control group (*p* < 0.000); see Additional files 1, 2, 3. Further we saw that companies in general did not impact the study duration significantly (− 0.002, *p* = 0.314) (Table 5). However, differentiating by all top 30 pharma companies we saw that a large fraction of the investigated companies, e.g., Boehringer Ingelheim (− 0.45, *p* = 0.004), Merck KGaA (− 0.55, *p* = 0.006), and Roche (− 0.30, *p* = 0.038) had significantly lower study durations compared to our control group (AbbVie). The results suggest that different companies impact the study duration to different extents. While GlaxoSmithKline was 1.06 years faster in their clinical studies than AbbVie (*p* < 0.000), Amgen was only 0.30 years faster than AbbVie (*p* = 0.092) (see Additional files 1, 2, 3).

**Table 5:** Regression results

Study duration	Coefficient	SE	95% Conf. Intervall		*p*-value
Indication areas	0.169	0.004	0.161	0.178	0.000
Company	− 0.002	0.002	− 0.006	0.002	0.314
_cons	0.722	0.054	0.615	0.829	0.000

## Discussion

In this study, we analyzed the dynamics of pipeline progress and portfolio management in the pharmaceutical industry. We looked at conducted clinical trials and study durations of the top 30 pharmaceutical companies over the past two decades. We saw that the amount of conducted clinical trials increased until 2014 and decreased from then on across all indication areas. We generally found that pipeline progress and portfolio management differ widely between companies and over the years. Moreover, we saw that study phases were positively correlated with the study duration, i.e., the higher the study phase, the higher the study duration, and that different indication areas influence the study duration to different extents.

Although, this is—to the best of the authors’ knowledge—the first study to give an extensive overview of the pipeline progress and study durations, our results are in accordance with related literature. For example, prior studies showed that the majority of clinical trials are based on anti-cancer pharmaceuticals [8]. We can confirm this with our results. Out of 13,589 investigated clinical trials, 2720 clinical trials—and with that the highest amount of clinical trials in one indication area—were conducted in the ONCIM indication area (20.01%).

Also, it is well known that clinical trial phases can differ in length [23]. Our results support this. We saw that phase 1 clinical trials took on average 1.04 years to complete, while phase 2 and phase 3 clinical trials took substantially longer (phase 2: 2.32 years, phase 3: 2.56 years). Concluding, drugs in clinical trials of the 30 largest pharmaceutical companies take on average a combined 5.92 years to reach regulatory approval (after phase 3). This is very fast, considering that the three phases of clinical studies can sometimes take 10–15 years [24].

While the amount of conducted clinical studies per indication says a lot about the research focus of the pharmaceutical industry [25, 26], the decrease in clinical studies from 2014 on does not necessarily mean worse research. It may also be that companies might be simply focusing on the most promising drugs and do not put money into studies that are not promising or where the product does not make it to the regulatory approval stage and market access. In addition, more and more new health technology assessments (HTA)—which can be seen as a form of stricter regulation—have been introduced in several countries over the last years [27–29]. This could also have an impact on the pipeline and portfolio management of pharma companies and reduce the amount of conducted clinical trials due to stricter regulation and the fear of having wasted capacities if the new drug does not make it to the regulatory approval stage. Prior research also suggests that the big pharma model is transitioning to a leaner, more focused enterprise [4]. However, investigating the pipeline and portfolio of the top 30 pharma companies we cannot confirm this trend. Rather we see that the biggest companies try to keep their portfolio as large and diversified as possible and develop new pharmaceuticals across several indications (e.g., AstraZeneca: cardiovascular, renal and metabolic, oncology, respiratory, inflammation and autoimmune, neurological, infections and vaccines).

The extent to which different indications influence the duration of clinical trials is as yet under-researched. However, few papers have looked at study durations of single indications and found different study lengths [30–32]. With our results, we were able to confirm this once again, across all 14 ATC indication areas (e.g., ONCIM clinical studies took 3.27 years longer (*p* < 0.000) compared to healthy individuals/phase 1 clinical studies, SENS clinical studies only took 0.56 years longer than our control group (*p* < 0.000)).

Larger companies usually offer a larger portfolio, also across indications [18, 22]. As a result, these companies also conduct more clinical studies than smaller companies. We see this in our analyses as well, e.g., GSK conducted eight times as many clinical trials as Eisai. Smaller companies often tend to focus on a few indications. However, this does not mean that they also achieve shorter study durations, e.g., GSK on average 1.93 years per clinical study vs. Eisai 2.53 years per clinical study. However, one advantage of smaller companies is that they are often more agile and flexible because they do not have such strict internal rules and structures as large companies [33, 34]. This means that they can still carry out their clinical studies quickly, when put into relation.

One of the core functions in all companies is to make effective decisions in a timely manner. Drug development is particularly closely linked to timing, as financial constraints are closely related to the different phases of drug development, each of which has specific funding needs [35]. How pharmaceutical companies build their pipeline and portfolio will be seen in the future. Importantly, decision-making here is not a static process, but rather a dynamic one that can change over time [36]. Previous research models have already developed an analytical decision-making tool to assess and improve a company's global portfolio while balancing business needs with broader societal expectations [37].

Future research could look at how new health technology assessment (HTA) regulations affect the pipeline progress, study duration, and portfolio management in the pharmaceutical industry. Also, it could be investigated for which products pharmaceutical companies conduct voluntary real-world evidence studies (e.g., only for products for which high sales are expected or for high topic indications like oncology).

### Limitations

Our analyses have several limitations. First, while we have collected and analyzed the data for the top 30 pharma companies, the results for smaller pharma companies could of course be different. However, smaller pharma companies also do not conduct as many clinical studies as larger companies do. Therefore, we cannot make any general statement about the pipeline progress and portfolio management for substantially smaller pharma companies.

Second, while we have accurately screened clinicaltrials.gov for all clinical studies from the top 30 pharmaceutical companies, we may not have found all clinical studies or some clinical studies may not have been listed on clinicaltrials.gov. Accordingly, we could only examine the studies that were available in the database.

Third, our calculations of study duration can only be based on available data. While for some clinical studies exact starting dates were reported, others have only the starting month and the year, which could have impacted our results slightly. Also, our descriptive results are based on means which implies that potential outliers could have caused the mean to go up or down. However, as the results across indications are in accordance with related literature, bias are expected to be rather small.

Fourth, our analyses are based on existing data from the past two decades (2000–2020). We cannot make any statement about how the pipeline progress and portfolio management will change in the future. However, our analyses are not intended to serve as a forecast and are rather intended to represent the pipeline progress and portfolio management in the pharma market in the past 20 years.

Lastly, two researchers independently reviewed all the variables in the database several times. Although carried out to the best of our abilities, manual checks are always accompanied by uncertainties. However, because two reviewers independently performed the check, errors should have been reduced to a minimum.

## Conclusion

With this research paper, we provide an extensive overview of the pipeline progress and study durations of the top 30 pharma companies worldwide over two decades (2000–2020). We found that pipeline progress and portfolio management differ widely between companies and over the years. Most of the clinical studies were conducted in the areas of ONCIM (*N* = 2720), META (*N* = 1993), NEURO (*N* = 1716), and INFEC (*N* = 1364). The indication with the highest study duration was ONCIM (on average 3.9 years per clinical study). Indications with the lowest study duration were SENS (on average 1.1 years per clinical study) and RESP (on average 1.5 years per clinical study). Values for the study duration vary widely across companies. Mostly they range between 1–4 years (e.g., GlaxoSmithKline on average 1.9 years per clinical study, Merck Sharp & Dohme (MSD) on average 2.2 years per clinical study, Novartis on average 2.9 years per clinical study). Correlation analysis using Spearman’s rank correlation coefficient showed that study phases were positively correlated with the study duration (0.36, *p* < 0.000), i.e., the higher the study phase, the higher the study duration. Furthermore, we found that indication areas influenced the study duration significantly (+ 0.169, *p* < 0.000). However, there were wide variations in effect sizes. While ONCIM clinical studies took 3.27 years longer (*p* < 0.000) compared to healthy individuals/phase 1 clinical studies, SENS clinical studies only took 0.56 years longer than our control group (*p* < 0.000). The results suggest that different indication areas influence the study duration to different extents.

Research findings could help portfolio managers to promote current indication areas of strong research and to better estimate the time to approval and thus allocate their company’s resources more efficiently. In this way, market and resource perspectives can be interlinked and, in addition, a development forecast of the respective portfolio dimensions can be made. By doing that, corporate strategy managers would be able to make more informed decisions regarding their business development strategy.

### Supplementary Information

**Additional file 1.** Indication Areas, ATC code level 1. 

**Additional file 2.** Clinical trials (N) per study phase over the past two decades.

**Additional file 3.** Regression results, Impact of indication areas and companies on study duration.

## Data Availability

Data sharing is not applicable to this article as no datasets were generated or analyzed during the current study.
